# Regions identity between the genome of vertebrates and non-retroviral families of insect viruses

**DOI:** 10.1186/1743-422X-8-511

**Published:** 2011-11-10

**Authors:** Gaowei Fan, Jinming Li

**Affiliations:** 1 National Center for Clinical Laboratories, Beijing Hospital, Beijing 100730, China; 2 Graduate School, Peking Union Medical College, Chinese Academy of Medical Sciences, Beijing, 100730, China

## Abstract

**Background:**

The scope of our understanding of the evolutionary history between viruses and animals is limited. The fact that the recent availability of many complete insect virus genomes and vertebrate genomes as well as the ability to screen these sequences makes it possible to gain a new perspective insight into the evolutionary interaction between insect viruses and vertebrates. This study is to determine the possibility of existence of sequence identity between the genomes of insect viruses and vertebrates, attempt to explain this phenomenon in term of genetic mobile element, and try to investigate the evolutionary relationship between these short regions of identity among these species.

**Results:**

Some of studied insect viruses contain variable numbers of short regions of sequence identity to the genomes of vertebrate with nucleotide sequence length from 28 bp to 124 bp. They are found to locate in multiple sites of the vertebrate genomes. The ontology of animal genes with identical regions involves in several processes including chromatin remodeling, regulation of apoptosis, signaling pathway, nerve system development and some enzyme-like catalysis. Phylogenetic analysis reveals that at least some short regions of sequence identity in the genomes of vertebrate are derived the ancestral of insect viruses.

**Conclusion:**

Short regions of sequence identity were found in the vertebrates and insect viruses. These sequences played an important role not only in the long-term evolution of vertebrates, but also in promotion of insect virus. This typical win-win strategy may come from natural selection.

## Background

The interaction between viruses and animals is quite profound and complex. Precious studies have deeply increased the depth of our understanding of their long-term evolutionary history in terms of genome sequence. Viruses have a highly host-associated life circle. As a result, they infect and occasionally integrate into the germ line cells chromosome and are inherited vertically as host alleles [1,2]. A growing number of nucleotide sequences of viruses have been and continue to be found in their respective host spices. These remnants of ancient viral infections play an important role in offering not only unforeseen sources of genomic novelty in their hosts [1,3] but also molecular fossils to facilitate our knowledge of the evolution process between viruses and animals [4]. Some of these sequences identity in host species were found to highlight several pathways including cell adhesion, Wnt signalling[5] and immunomodulation [6] as well as mammalian reproduction [7].

However, most of these discoveries were merely addressed in an aspect of virus-host interaction and may narrow our prospective to probe the links between viruses and animals.

Here in a broad sense, we aimed at to identify the possible regions identity between the genomes of vertebrates and non-retroviral families of insect viruses and the possible role(s) of the identical sequences in evolution of the corresponding animal(s). Moreover, we reported phylogenetic analysis of these identical sequences. In this paper, we showed that at least some of the sequences identity in vertebrates chromosomes identified here are likely to come from insect viruses and exapted during their long-term evolution.

## Results

We screened several hundreds of insect viruses including DNA viruses and RNA viruses against 21 vertebrates. Of interest, dozens of short regions of sequence identity were found between animals and viruses including double stranded DNA viruses and double stranded RNA viruses (Table 1). Note that in our study more short regions of sequence identity to a DNA-virus were found than that to a RNA-virus which was also reported in precious study [8]. Ranging from 28 bp to 124 bp, these sequences identity were found in two possible orientations in the respective animals. Most of these regions were found in intergenic regions of the genomes, some were within introns. However, with occasional exception, regions of identity were also found within gene-coding region. For example, in the case of duck-billed platypus, sequence identity to Phthorimaea operculella granulovirus occured within exon and coded protein similar to ubiquitin. Pieces of sequence identity that copy themselves and reinsert into the genome of animals could be found in our study. Besides, two distinct short regions of sequence identity to a certain virus also occurred in the same genome of the animal suggesting that more than one distinct short region derived from a virus invaded and fixed into the same animal genome. For example, in the case of zebra finch two distinct short regions identity to Choristoneura occidentalis granulovirus were found within the genome [GenBank:NW_002197778.1] with respective E-values 4e-23 and 1e-14.

**Table 1:** Insect viruses and vertebrates sequences, showing the regions of sequence identity (> = 28 bp)

Virus Family	Virus name	accession number	Animal name	GenBank accession number	Nucleotide position of animals	Length (bp)	E-value	Identity
Baculoviridae	CuniNPV	NC_003084	Mouse	NW_001030773.1	2590199-2590237	39	1e-4	95%
			Rat	NW_047339.1	3135495-3135526	32	4e-4	100%
				NW_001084656.1	73274015-73274046	32	4e-4	100%
	ChocGV	NC_008168	Chicken	NW_001471682.1	139049-139125	77	8e-20	92%
				NW_001471533.1	8588-8682	59	8e-20	89%
					11486-11562	77	4e-18	91%
			western clawed frog	NW_003173493.1	56-150	95	5e-23	91%
				NW_003170485.1	154-248	95	5e-23	91%
				NW_003170383.1	2710-2804	95	5e-23	91%
				NW_003167681.1	7042-7136	95	5e-23	91%
				NW_003165127.1	14113-14207	95	5e-23	91%
				NW_003164260.1	65463-65557	95	5e-23	91%
				NW_003170529.1	87-180	95	2e-21	89%
					2395-2471	77	5e-18	91%
				NW_003168613.1	7284-7360	77	5e-18	91%
			zebra finch	NW_002197778.1	10813-10907	95	4e-23	91%
					881-957	78	1e-14	88%
				NW_002222303.1	4415-4509	95	2e-21	89%
				NW_002221112.1	5155-5249	95	2e-21	89%
				NW_002205312.1	1925-2019	95	2e-21	89%
				NW_002197777.1	5158-5252	95	2e-21	89%
				NW_002215575.1	2342-2433	92	4e-18	88%
	CpGV	NC_002816	Opossum	NW_001583776.1	49362-49401	40	0.001	93%
			Rat	NW_047512.2	4126149-4126187	39	1e-4	95%
				NW_001084735.1	26022662-26022700	39	1e-4	95%
				NW_047471.2	4060358-4060389	32	5e-4	100%
				NW_047801.1	6211322-6211356	35	5e-4	97%
				NW_001084717.1	537999-538030	32	5e-4	100%
				NW_001084876.1	27348810-27348844	35	5e-4	97%
			Rabbit	NW_003159328.1	7491714-7491744	31	9e-4	100%
	ChchNPV	NC_007151	Sumatran orang-utan	NW_002879912.1	596051-596082	32	4e-4	100%
	EcobNPV	NC_008586	mouse	NT_039649.7	55558397-55558431	35	5e-4	97%
			Marmoset	NW_003184594.1	7284126-7284248	124	4e-18	82%
			Rat	NW_047689.2	35310450-35310491	35	5e-4	97%
				NW_001084827.1	32823677-32823711	35	5e-4	97%
	SfMNPV	NC_009011	Zebrafish	NW_001878120.3	69811-69844	35	5e-4	97%
	SeMNPV	NC_002169	Rat	NW_047356.1	22195469-22195510	43	2e-4	93%
				NW_001084662.1	30770186-30770227	43	2e-4	93%
	LsNPV	NC_008348.1	Rhesus macaque	NW_001105692.1	773081-773115	35	4e-4	97%
			Opossum	NW_001582020.1	41004052-41004090	39	1e-4	95%
			Panda	NW_003218644.1	263401-263436	36	8e-5	97%
			Rat	NW_047829.1	1021494-1021528	35	7e-4	97%
				NW_047473.1	17381564-17381598	35	7e-4	97%
				NW_047711.2	25617419-25617456	38	7e-4	95%
				NW_001085491.1	25617419-25617456	35	7e-4	97%
				NW_001084718.1	16637290-16637324	35	7e-4	97%
				NW_001084836.1	24037274-24037311	38	7e-4	95%
	MaviMNPV	NC_008725	Marmoset	NW_003183861.1	808726-808762	38	9e-4	95%
	PoGV	NC_004062	duck-billed platypus	NW_001794503.1	629177-629241	67	2e-4	84%
	AdorGV	NC_011423	Rabbit	NW_003159291.1	19601679-19601713	35	2e-4	97%
				NW_003159237.1	33249365-33249399	35	2e-4	97%
				NW_003159323.1	30674664-30674697	34	8e-4	97%
				NW_003159357.1	771225-771258	34	8e-4	97%
	GflV	NC_008923	Zebrafish	NW_001878107.3	889847-889879	33	2e-4	97%
					889234-889261	28	7e-4	100%
				NW_001877665.3	641091-641118	28	7e-4	100%
					965204-965231	28	7e-4	100%
Ascoviridae	TnAV-2c	NC_008518	Sumatran orang-utan	NW_002880108.1	598679-598717	39	4e-4	95%
Polydnaviridae	MdBV	NC_007034	Marmoset	NW_003184482.1	2186961-2186992	32	2e-5	100%
				NW_003184465.1	644797-644825	29	0.001	100%
	HfIV	NC_008949	Rhesus macaque	NW_001124102.1	1986281-1986308	28	0.001	100%
	CcBV	NC_006651	Duck-billed platypus	NW_001753059.1	454802-454833	32	0.001	97%
		NC_006654	Western clawed frog	NW_003163742.1	13336070-13336110	30	2e-4	100%
		NC_006646	Rat	NW_047553.1	6590148-6590200	53	6e-8	91%
				NW_001084751.1	4855654-4855706	53	6e-8	91%
	CsIV	NC_007989	Zebra finch	NW_002198116.1	15663-15691	29	4e-4	100%
				NW_002198283.1	2552721-2552749	29	4e-4	100%
Reoviridae	NLRV	NC_003659.1	Zebrafish	NW_001877567.3	335436-335467	32	5e-4	97%
	FDV	NC_007155	Duck-billed platypus	NW_001794408.1	3462447-3462475	29	2e-4	100%
	RBSDV	NC_014709	Horse	NW_001867377.1	20656389-20656416	28	8e-4	100%

### The relationship between pseudo-genes and sequences identity

The phenomenon that a large number of identified regions were located near or within pseudo-genes caused our attention and promoted us to investigate what the relationship between the sequences identity and pseudo-genes was. To investigate this phenomenon further, we calculated the distance between the pseudo-genes and the end(s) of the regions identity as described in Methods.

**Figure 1:**
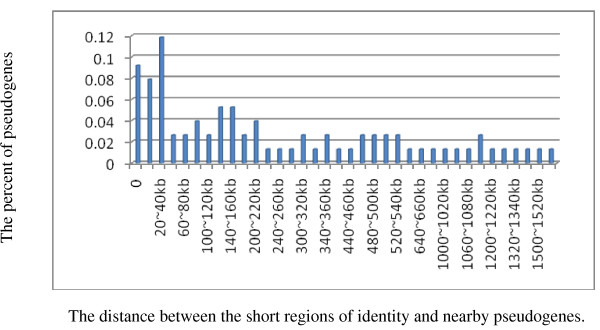
**The relationship between sequences identity and rate of nearby pseudo-gene**.

Figure 1 shows the relationship between the distance from the ends of a short region of identity to the related pseudo-gene and the percentage of pseudo-gene within the distance. In our study, 7 out of 76 pseudo-genes harbor short regions of sequence identity. A rough rule of the distribution is that most of the pseudo-genes are within 1000 kb flanking the ends of the short regions of identity.

### Roles of genes containing sequences identity

Table 2 shows the important roles of genes containing regions of sequence identity play in the evolution of vertebrates ranging from chromatin remodeling, mitotic cell cycle, signaling pathway, gene switch to signal transduction, cell-cell adhesion and nervous system development.

**Table 2:** Biological process or molecular function of the regions of sequence identity products.

Virus sp	GenBank accession no.	Animal name	GenBank accession no.	Location	Gene products	Gen ID	Biological Process or Molecular Function of products
CuniNPV	NC_003084	Mouse	NW_001030773.1	intron	Rere	68703	Chromatin remodeling; multicellular orga ismal development; regulation of transcription, DNA- dependent
		Rat	NW_047339.1	intron	Psme3	287716	amine metabolic process; oxidation-reduction
		Rat	NW_001084656.1	intron	Psme3	287716	process; regulation of ubiquitin-protein ligase activity involved in mitotic cell cycle; positive regulation of endopeptidase activity; involved in mitotic cell cycle
ChocGV	NC_008168	Chicken	NW_001471533.1	intron	similar to neuropeptide	769505	some enzyme-like catalysis; many are involved
		Western clawed frog	NW_003170485.1	intron	miscRNA	100487653	in processing RNA after it is formed; some
		Western clawed frog	NW_003170529.1	intron	miscRNA	100488236	of these small RNAs may serve as switches, turning genes on and off; RNAi, silence genes by tagging their mRNA for destruction
CpGV	NC_002816	Rat	NW_047471.2	intron	Sh3rf1	306417	zinc ion binding
		Rat	NW_001084717.1	intron	Sh3rf1	306417	
		Rat	NW_047801.1	intron	Ephb1	24338	axon guidance; camera-type eye morphogenesis;
		Rat	NW_001084876.1	intron	Ephb1	24338	central nervous system projection neuron axonogenesis; cranial nerve axonogenesis; development; optic nerve morphogenesis; retinal ganglion cell axon guidance; signal transduction; transmembrane receptor protein tyrosine kinase signaling pathway
EcobNPV	NC_008586	Marmoset	NW_003184594.1	intron	ZC3H11A	100412100	Metal ion binding; nucleic acid binding; protein binding; zinc ion binding
LsNPV	NC_008348	Rhesus macaque	NW_001105692.1	intron	NFATC1	698089	Calcium ion transport; epithelial to mesenchymal transition; G1/S transition of mitotic cell cycle; heart development; intracellular signal transduction; positive regulation of transcription from RNA polymeraseII promoter; regulation of transcription, DNA-dependent
		Panda	NW_003218644.1	intron	CNTN3	100473475	cell-cell adhesion; nervous system development
PoGV	NC_004062	Duck-billed platypus	NW_001794503.1	exon	similar to ubiquitin	100078088	
CsIV	NC_007989	Zebra finch	NW_002198116.1	intron	similar to protein phosphatase 1	100221684	
NLRV	NC_003659	Zebrafish	NW_001877567.3	intron	suppressor of tumori- genicity14 protein homolog	557248	peptidolysis
FDV	NC_007155	Duck-billed platypus	NW_001794408	intron	similar to 145 kDa - nucleolar protein	100084172	
RBSDV	NC_014709	Horse	NW_001867377.1	intron	similar to early B-cell factor 1 isoform 3	100059840	

### Phyogenetic analysis

A screen of vertebrate genomes has unexpectedly exhumed short regions of sequence identity to insect viruses leading us to speculate about the evolutionary relationship among these sequences. And then phyogenetic comparisons of these sequences identity were performed as described in Methods.

#### Sequence identity to Adoxophyes orana NPV

**Figure 2:**
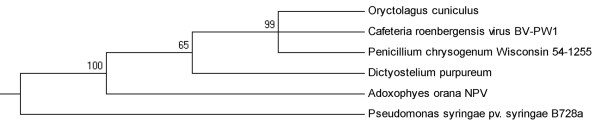
**Phylogenetic relationship of short regions of identity to Adoxophyes orana NPV**.

Significant blast hits to Adoxophyes orana NPV were sequences from species including mammalian, virus, fungi and bacteria (Figure 2). Sequences from Oryctolagus cuniculus, Cafeteria roenhergensis virus BV-PW1, Penicillium chrysogenum Wisconsin 54-1255, Dictyostelium purpureum and Adoxophyes orana NPV grouped into a single group with robust bootstrap support (100%), suggesting that they are likely derived from the same lineage. Cafeteria roenhergensis virus has the largest genome of any described marine virus and infects a widespread marine phagocytic protest [9]. The argument that cafeteria roenhergensis virus belongs to the fourth domain of life is supported by recent study [10].

#### Sequence identity to Choristoneura occidentalis granulovirus

**Figure 3:**
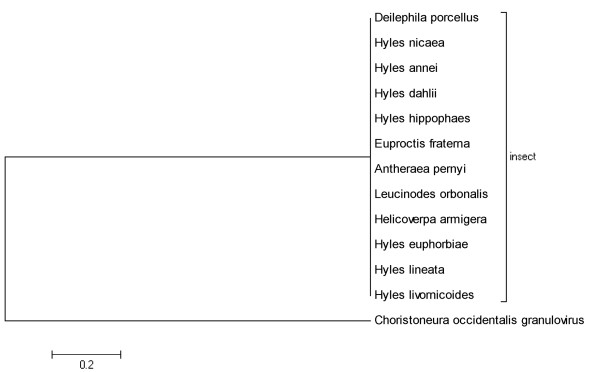
**Phylogenetic relationship of short regions of identity to Choristoneura occidentalis granulovirus**.

Sequences matching Choristoneura occidentalis granulovirus were all identified in insects (Figure 3). In phylogenies, these short regions identity grouped into two clades, the largest of which included matches related to insect genomes suggesting that they are from the same ancestral lineage. Sequence derived from Choristoneura occidentalis granulovirus formed a single clade. It's hard for us to know whether sequences from insects originated from distinct Choristoneura occidentalis granulovirus linage or not.

#### Sequence identity to Culex nigripalpus baculovirus

**Figure 4:**
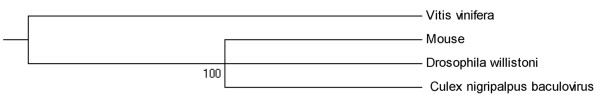
**Phylogenetic relationship of short regions of identity to Culex nigripalpus baculovirus**.

We identified high-level significant matches to Culex nigripalpus baculovirus in the genomes of plant, mammalian, insect (Figure 4). Phylogenies constructed grouped Mouse, Drosophila willistoni with Culex nigripalpus baculovirus with a robust support (100%), suggesting they are likely derived from the same exogenous lineage.

#### Sequence identity to Cydia pomonella granulovirus

**Figure 5:**
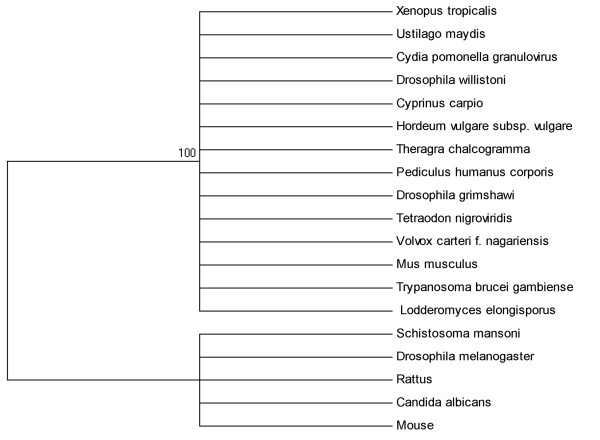
**Phylogenetic relationship of short regions of identity to Cydia pomonella granulovirus**.

Significant matches to Cydia pomonella granulovirus are short regions identified in a broad range of lineage genomes including chordate, fungi, insects, vertebrates, protozoa and plant (Figure 5). Curiously, Cyprinus carpio, Mus musculus and Theragra chalcogramma and some other species grouped together into a larger well-surpported clade with Cydia pomonella granulovirus while Mouse, Rattus, Schistoroma mansoni and Drosophila melanogaster as well as Candida albicans grouped into a smaller clade. Considering that a closely related species doesn't group into the same clade, the initial nucleotide sequences flow from Cydia pomonella to the ancestor of the Mus musculus at least post dated the split of Mus musculus and Rattus norvegicus which occurred about 10 million years ago [11].

#### Sequence identity to Leucania separata

**Figure 6:**
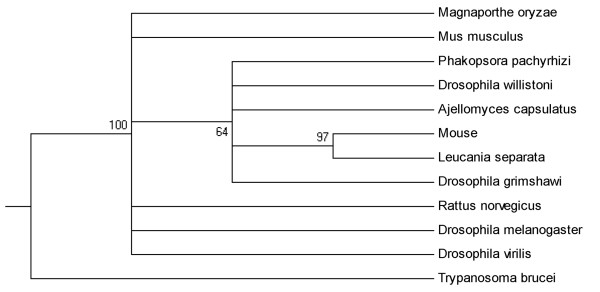
**Phylogenetic relationship of short regions of identity to Leucania separate**.

Matches to Leucania separata were sequences from different species ranging from fungi, mammalians, bacteria and protozoa as well as insects (Figure 6). Interestingly, with a robust bootstrap support (97%) sequences from Mouse and Leucania separata grouped into a single group suggesting that they are likely derived from the same ancestral lineage. As for sequences identity from Mus musculus, Rattus norvegicus, fungi and bacteria they may derive from distinct Leucania separata lineages.

## Discussion

In order to broaden the scope of people's understanding of the interaction between virus and animals, We searched genomes of 21 currently available vertebrates for sequences identity to that of insect viruses with expectation that possible sequences identity may exist, and unearthed lush short regions of sequence identity in diverse animals. The chance matches of the search were ruled out by performing reciprocal BLAST. With sequence length from 28 to 124 bp, most of them are non-functional, however, with exceptional occasions, some are within exon.

The mechanism that nucleotide sequences flowed from ancestral insect viruses to vertebrates is still unclear. A possible explanation for the phenomenon is due to genetic mobile element such as virus and phage as well as plasmid. Earlier study shows that viruses move between different biomes and the total number of viruses largely exceeds the number of cells [12]. In our data, short regions of sequence identity to virus is also found in bacteria, for example, in the case of Leucania separata, short region of identity is found in Ajellomyces capsulatus. Besides, short regions of sequence identity in the genomes of bacteria and bacteriophages as well as human were identified recently [13]. And further study is still warranted.

The fate of most acquired nucleotide sequences in the chromosomes of animals has been to undergo deletion due to homologous recombination [14], however, the deletion rate decreased dramatically with age [14], and finally only few fragments of the sequences fixed into the genomes of germ line cells and passed from parent to offspring vertically. These obtained sequences undoubtedly play a pivotal role in shaping vertebrates genome. Among the products of the short regions of sequence identity, some involve in interaction with animals: chromatin remodeling, regulation of apoptosis, signaling pathway, nerve system development and some enzyme-like catalysis. On one hand, these products take in part in the formation of vertebrate, help to promote the evolution of vertebrates. On the other hand, likewise, these products play an important role in promotion of virus persistence [5,15]. For the survival of virus, the ideal can be achieved that the impact of its infection will not harm the host and the risk of host pathology will be reduced with a long-term host [15]. From this aspect, the phenomenon that virus invaded animal(s) and fixed its nucleotide sequences into the genomes of the germ line cells and passed vertically is a typical win-win strategy both for the survival of virus sequences and the long-term evolution of animal(s).

No discussion of short regions of sequence identity would be complete without mention pseudo-genes. Pseudo-gene which is known for non-functional, gene-like sequences due to a high mutation rate is harbored by mammalian genomes [16]. Lacking functional promoters or other regulatory elements, a pseudo-gene is not transcribed [17,18]. Coincide with the studies that a fixed viral insertion possibly decay into a pseudo-gene [1,17], in our study 7 out of 76 pseudo-genes harbor short regions of sequence identity. However, it is quite confused that dozens of pseudo-genes were located near the short regions identity from several hundred base pair to more than one million base pair. A rough rule is that most of them are within 1 Mb. The reason why so many pseudo-genes are located nearby is not clear. The explanation that the distribution of nearby pseudo-genes is by chance seems not likely. The fact that pseudo-genes tend to occur in the genome of families with environmental-response functions shows that instead of being dead, they may form a reservoir of diverse "extra part" which can be helpful for an organism to get used to its surroundings [19]. Alternative explanation is that the short regions of sequence identity may function by an unknown regulatory mechanism in the formation of pseudo-genes. Note that in our study, in the case of western clawed frog, short regions identity to Choristoneura occidentalis granulovirus were within intron of the gene whose product is miscRNA. MiscRNA is short for miscellaneous RNA, a general term for a series of miscellaneous small RNA. It serves a variety of functions, including some enzyme-like catalysis and processing RNA after it is formed. Besides, some of these small RNAs may serve as switches. Others, called RNAi, silence genes by tagging their mRNA for destruction [20,21]. Maybe some of these small RNAs serve as gene switches, turning genes on and off, or just silence genes with the help of RNAi. Besides, it's known that enhancers as well as other regulatory elements can be 1 Mb from the target gene [22]. The phenomenon that most nearby pseudo-genes are within 1 Mb coincides with the description above. Apparently, further study is needed to address this possibility.

We have investigated the evolutionary radiation of some of the identified short regions of insect viruses and demonstrated a broad history of interaction between insect viruses and vertebrates. It is interesting to speculate that short regions of identity occurred across a brand species. According to our data, at least some short regions of identity identified in vertebrates are derived from insect viruses. And the initial gene flow from Cydia pomonella to the ancestor of the Mus musculus at least post dated the divergence of Mus musculus and Rattus norvegicus about 10 million years ago. However, due to the limited samples, it is hard for us to know whether some sequences identity of the insect viruses and that of vertebrates shared the same ancestral lineage or not. Since the evolution of some viral sequences is more rapid than that of animals, it may mask any two nucleotide sequences which actually derived from the same ancestor [23].

## Conclusions

Our study established that the genetic material derived from insect viruses can flow to vertebrates and play a significant evolutionary role for the development of vertebrates and the survival of the viruses. This win-win strategy may be the result of natural selection.

## Methods

### Genome screening

The genomes of non-retroviral families of insect viruses were screened against chromosome assemblies and whole genome shotgun assemblies of 21 vertebrate species in silico approach using BLASTn with the resources of NCBI. Insect viruses sequences with a high-level identity (i.e. e-value < 0.001) of matches to vertebrates nucleotide sequences were acquired. Then the acquired animal sequences were used as queries to screen the GenBank non-redundant (nr) database in a reciprocal BLASTn search. Significant matches to retroviruses and non-insect viruses were discarded, while the remaining matches were considered as regions of identity to non-retroviral families of insect viruses.

Regions of identity were located in corresponding genome shotgun assemblies of vertebrates precisely. If pseudo-genes were found near regions of identity (i.e. 2000 kb within their 5' and/or 3' ends) distance was calculated between the nearby pseudo- genes and 5'site and/or 3's site of regions of identity.

### Phylogenetic analysis

For understanding the distribution and possible origin of sequences identity, BLASTn was run with virus sequences as queries to screen the GenBank non-redundant (nr) database. Significant hits with over 95% identity and blast E-values of 10-7 or lower were identified as regions of sequence identity. And representative sequences were extracted. These nucleotide sequences were aligned using ClustalX[24] program and manually edited. Neighbor-Joining (NJ) phylogenies[25] were then constructed using the nucleotide sequence alignments with PHYLIP [26]. A consensus tree was calculated with the program Consensus of the PHYLIP package. Support for the ML trees was evaluated with a total of 1,000 bootstrap replicates.

### Vertebrate name

Mammals: Primates (= 5): Callithrix jacchus (white-tufted-ear marmoset); Homo sapiens (human); Macaca mulatta (rhesus macaque); Pan troglodytes (chimpanzee); Pongo abelii (Sumatran orangutan); Rodents (= 2): Mus musculus (laboratory mouse); Rattus norrvegicus (rat)

Monotremes (= 1): Ornithorhynchus anatinus (duck-billed platypus) Marsupials (= 1):Monodelphis domestica (opossum) Other Mammals (= 8): Ailuropoda melanoleuca (giant panda); Bos taurus (cattle); Canis lupus familiaris (dog); Equus caballus (horse); Felis catus (cat); Oryctolagus cunniculus (rabbit); Ovis aries (sheep); Sus scrofa (pig) Other Vertebrates (= 4): Danio rerio (zebrafish); Gallus gallus (chicken); Taeniopygia guttata (zebra finch); Xenopus tropicalis (Silurana) (western clawed frog)

### Sequences and accession numbers of insect viruses

Baculoviridae: Choristoneura fumiferana DEF MNPV [GenBank:NC_005137]; Agrotis segetum granulovirus [GenBank:NC_005839]; Helicoverpa armigera NPV G4 [GenBank:NC_002654]; Orgyia pseudotsugata MNPV [GenBank:NC_001875]; Mamestra configurata NPV-A [GenBank:NC_003529]; Cydia pomonella granulovirus [GenBank:NC_002816]; Spodoptera exigua MNPV [GenBank:NC_002169]; Bombyx mori NPV [GenBank:NC_001962]; Bombyx mandarina NPV [GenBank:NC_012672]; Spodoptera frugiperda MNPV virus [GenBank:NC_009011]; Lymantria xylina MNPV [GenBank:NC_013953]; Mamestra configurata NPV-B [GenBank:NC_004117]; Lymantria dispar MNPV[GenBank:NC_001973]; Epiphyas postvittana NPV[GenBank:NC_003083]; Xestia c-nigrum granulovirus [GenBank:NC_002331]; Autographa californica NPV [GenBank:NC_001623]; Helicoverpa armigera NPV NNg1[GenBank:NC_011354]; Pieris rapae granulovirus[GenBank:NC_013797]; Pseudaletia unipuncta granulovirus [GenBank:NC_013772]; Agrotis segetum NPV [GenBank:NC_007921]; Spodoptera litura granulovirus[GenBank:NC_009503]; Chrysodeixis chalcites NPV [GenBank:NC_007151]; Neodiprion abietis NPV [GenBank:NC_008252]; Neodiprion lecontii NPV[GenBank:NC_005906]; Cryptophlebia leucotreta granulovirus [GenBank:NC_005068]; Adoxophyes orana granulovirus[GenBank:NC_005038]; Helicoverpa armigera NPV [GenBank:NC_003094]; Rachiplusia ou MNPV[GenBank:NC_004323]; Phthorimaea operculella granulovirus [GenBank:NC_004062]; Spodoptera litura NPV [GenBank:NC_003102]; Culex nigripalpus NPV [GenBank:NC_003084]; Plutella xylostella granulovirus [GenBank:NC_002593]; Heliothis zea virus 1 [GenBank:NC_004156]; Clanis bilineata NPV [GenBank:NC_008293]; Neodiprion sertifer NPV [GenBank:NC_005905]; Trichoplusia ni SNPV [GenBank:NC_007383]; Choristoneura fumiferana MNPV [GenBank:NC_004778]; Helicoverpa zea SNPV [GenBank:NC_003349]; Euproctis pseudoconspersa NPV [GenBank:NC_012639]; Agrotis ipsilon multiple NPV [GenBank:NC_011345]; Orgyia leucostigma NPV [GenBank:NC_010276]; Helicoverpa armigera granulovirus [GenBank:NC_010240]; Ecotropis obliqua NPV [GenBank:NC_008586]; Anticarsia gemmatalis NPV [GenBank:NC_008520]; Choristoneura occidentalis granulovirus [GenBank:NC_008168]; Adoxophyes honmai NPV [GenBank:NC_004690]; Hyphantria cunea NPV [GenBank:NC_007767]; Antheraea pernyi NPV [GenBank:NC_008035]; Spodoptera litura nucleopolyhedrovirus II [GenBank:NC_011616]; Helicoverpa armigera multiple NPV [GenBank:NC_011615]; Adoxophyes orana NPV [GenBank:NC_011423]; Maruca vitrata MNPV [GenBank:NC_008725]; Plutella xylostella multiple NPV [GenBank:NC_008349]; Leucania separata nuclear polyhedrosis virus [GenBank:NC_008348]

Entomopoxvirinae: Amsacta moorei entomopoxvirus 'L' [GenBank:NC_002520]; Melanoplus sanguinipes entomopoxvirus [GenBank:NC_001993] Ascoviridae: Spodoptera frugiperda ascovirus 1a [GenBank:NC_008361]; Diadromus pulchellus ascovirus 4a [GenBank:NC_011335]; Heliothis virescens ascovirus 3e [GenBank:NC_009233]; Trichoplusia ni ascovirus 2c [GenBank:NC_008518] Polydnaviridae: Hyposoter fugitivus ichnovirus [GenBank:NC_008946~ NC_008973, NC_008973~ NC_009003]; Microplitis demolitor bracovirus [GenBank: NC_007028 ~ NC_007041, NC_007044]; Cotesia congregata virus [GenBank:NC_006638~ NC_006640, NC_006649]; Cotesia congregata bracovirus [GenBank:NC_006633~ NC_006637, NC_006641~ NC_006645,NC_006647, NC_006648, NC_006650~ NC_006662]; Campoletis sonorensis ichnovirus [GenBank:NC_007985~ NC_008008]; Glypta fumiferanae ichnovirus [GenBank:NC_008837~ NC_008894, NC_008896~ NC_008910, NC_008912~ NC_008928]; Campoletis sonorensis ichnovirus [GenBank:NC_008006, NC_008895, NC_008911] Reoviridae: Southern rice black-streaked dwarf virus [GenBank:NC_014708~ NC_014717]; Great Island virus [GenBank:NC_014522~ NC_014531]; Stretch Lagoon orbivirus [GenBank:NC_012754, NC_012755]; Raspberry latent virus[GenBank: NC_014598~ NC_014607 ]; African horsesickness virus [GenBank:NC_005996, NC_006009, NC_006011, NC_006012, NC_006016~ NC_006021]; Epizootic hemorrhagic disease virus [GenBank:NC_013396~ NC_013405]; Kadipiro virus [GenBank:NC_004199, NC_004205~ NC_00421, NC_004212~ NC_004216]; Fiji disease virus [GenBank:NC_007154~ NC_007163]; St Croix River virus [GenBank:NC_005997~ NC_005998]; Operophtera brumata reovirus segment 1 [GenBank:NC_007559]; Mal de Rio Cuarto virus[GenBank:NC_008728~ NC_008737]; Eyach virus [GenBank:NC_003696~ NC_003707]; Aedes pseudoscutellaris reovirus [GenBank:NC_007666~ NC_007674]; Heliothis armigera cypovirus [GenBank:NC_010661~ NC_010670]; Yunnan orbivirus [GenBank:NC_007656~ NC_007665]; Rice ragged stunt virus [GenBank:NC_003749~ NC_003752, NC_003757~ NC_003759, NC_003769~NC_003771]; Nilaparvata lugens reovirus [GenBank:NC_003652~ NC_003661]; Trichoplusia ni cytoplasmic polyhedrosis virus [GenBank:NC_002557, NC_002559~ NC_002562, NC_002564~ NC_002567]; Homalodisca vitripennis reovirus [GenBank:NC_012535~ NC_012546]; Rice gall dwarf virus [GenBank:NC_009241~ NC_009252]; Banna virus [GenBank:NC_004198, NC_004200~ NC_004204]; Rice dwarf virus [GenBank:NC_003760~ NC_003768, NC_003772~ NC_003774 ]; Rice black streaked dwarf virus [GenBank:NC_003728~ NC_003737 ]; Lymantria dispar cypovirus1[GenBank:NC_003016~ NC_003025]; Cypovirus 14 [GenBank: NC_003006~ NC_003015] Birnaviridae: Drosophila x virus [GenBank:NC_004169, NC_004177] Dicistroviridae: Black queen cell virus [GenBank:NC_003784]; Triatoma virus [GenBank:NC_003783]; Drosophila C virus [GenBank:NC_001834]; Kashmir bee virus [GenBank:NC_004807]; Aphid lethal paralysis virus [GenBank:NC_004365]; Cricket paralysis virus [GenBank:NC_003924]; Rhopalosiphum padi virus [GenBank:NC_001874]; Israel acute paralysis virus of bees [GenBank:NC_009025]; Himetobi P virus [GenBank:NC_003782]; Acute bee paralysis virus [GenBank:NC_002548]; Plautia stali intestine virus [GenBank:NC_003779]; Solenopsis invicta virus 1 [GenBank:NC_006559]; Homalodisca coagulata virus-1 [GenBank:NC_008029] Tetraviridae: Euprosterna elaeasa virus [GenBank:NC_003412]; Boolarra virus [GenBank:NC_004142, NC_004145]; Pariacato virus chromosome [GenBank:NC_003691~ NC_003692]; Nodamura virus [GenBank:NC_002690~ NC_002691]; Black beetle [GenBank:NC_001411, NC_002037]; Macrobrachium rosenbergii nodavirus RNA-2 [GenBank:NC_005095]; Flock house virus [GenBank: NC_004146~ NC_004144 ]

## List of abbreviations used

ChchNPV: Chrysodeixis chalcites NPV; SfMNPV: Spodoptera frugiperda MNPV; SeMNPV: Spodoptera exigua MNPV; MaviMNPV: Maruca vitrata MNPV; AdorGV: Adoxophyes orana NPV; GflV: Glypta fumiferanae ichnovirus; TnAV-2c: Trichoplusia ni ascovirus 2c; MdBV: Microplitis demolitor bracovirus; HfIV: Hyposoter fugitivus ichnovirus; CcBV: Cotesia congregata bracovirus; CuniNPV: Culex nigripalpus NPV; ChocGV: Choristoneura occidentalis granulovirus; CpGV: Cydia pomonella granulovirus; EcobNPV: Ecotropis obliqua NPV; LsNPV: Leucania separata NPV; PoGV: Phthorimaea operculella granulovirus; CsIV: Campoletis sonorensis ichnovirus; NLRV: Nilaparvata lugens reovirus; FDV: Fiji disease virus; RBSDV: Southern rice black-streaked dwarf virus

## Competing interests

The authors declare that they have no competing interests.

## Authors' contributions

Conceived the experiment: JL, Designed the experiment: GF; Performed the experiment: GF; Analyzed the data: GF; Wrote the paper: GF; Revised the paper: JL; gave the final approval of the version to be published: JL. Both authors read and approved the final manuscript.
